# 1423. A Simple Electronic Medical Record-Based Predictors of Illness Severity in Sepsis (SEPSIS) Score

**DOI:** 10.1093/ofid/ofad500.1260

**Published:** 2023-11-27

**Authors:** Alex Cressman, Bijun Wen, Sudipta Saha, Hae Young Jun, Riley Waters, Sharan Lail, Aneela Jabeen, Radha Koppula, Lauren Lapointe-Shaw, Kathleen Sheehan, Nick Daneman, Amol Verma, Fahad Razak, Derek MacFadden

**Affiliations:** University of Toronto, Toronto, Ontario, Canada; Unity Health, Toronto, Ontario, Canada; Unity Health, Toronto, Ontario, Canada; Unity Health, Toronto, Ontario, Canada; Unity Health, Toronto, Ontario, Canada; Unity Health, Toronto, Ontario, Canada; Unity Health, Toronto, Ontario, Canada; Unity Health, Toronto, Ontario, Canada; University Health Network, Toronto, Ontario, Canada; University Health Network, Toronto, Ontario, Canada; Sunnybrook Health Sciences Centre, University of Toronto, Toronto, Ontario, Canada; Unity Health, Toronto, Ontario, Canada; Unity Health, Toronto, Ontario, Canada; The Ottawa Hospital Research Institute, Ottawa, Ontario, Canada

## Abstract

**Background:**

Current scores for predicting sepsis outcomes are limited by generalizability, complexity, and electronic medical record (EMR) integration. Here, we validate a simple EMR-based score for sepsis outcomes in a large multi-centre cohort.

**Methods:**

A simple electronic medical record-based predictor of illness severity in sepsis (SEPSIS) score was developed (4 additive lab-based predictors: Creatinine, Bilirubin, Platelet Count, Lactate) using a retrospective cohort study of patients admitted to internal medicine services from April 2010 - March 2015, across four hospitals in Toronto, Canada. We identified patients with sepsis based upon receipt of antibiotics and positive (non-screening) cultures on admission. Chart review was conducted to extract components of qSOFA and NEWS2 scores. The primary outcome was in-hospital mortality and secondary outcomes were ICU admission at 72 hours, and hospital length of stay (LOS). We calculated the area under the receiver operating curve (AUROC) for the SEPSIS score, qSOFA, and NEWS2. We then evaluated the SEPSIS score in a contemporary cohort (2015 to 2019) of patients receiving systemic antibiotics.

**Results:**

Our initial cohort included 1,890 patients with a median age of 72 years (IQR: 56-83). 9% of the cohort died during hospitalization, 13% were admitted to ICU at 72 hours, and mean LOS was 12.7 days (SD: 21.5). In the initial and the contemporary (2015-2019, 4811 patients) cohorts, the AUROCs of the SEPSIS score for predicting in-hospital mortality were 0.63 and 0.64 respectively, which were similar to NEWS2 (0.62 and 0.67) and qSOFA (0.62 and 0.68). AUROCs for predicting ICU admission at 72 hours, and length of stay >14 days, were similar between scores, in the initial and contemporary cohorts. All scores were generally well calibrated for predicting mortality (Figure 1).

**Figure 1.**
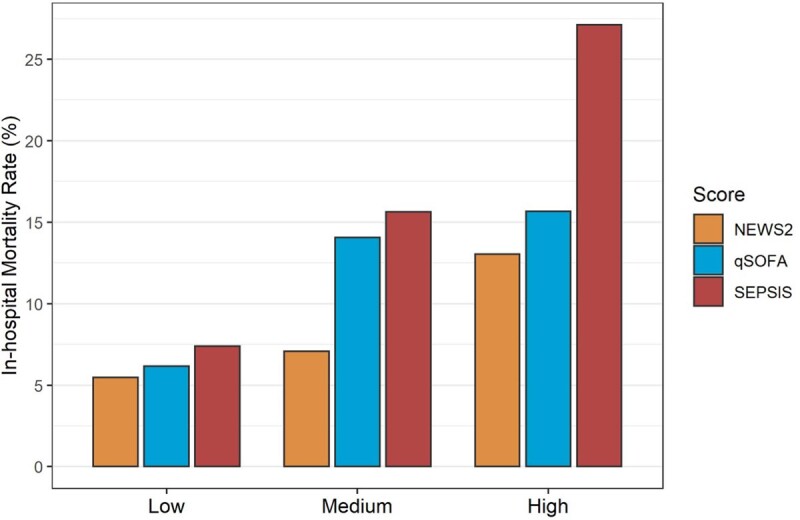
Mortality risk by predictor score (low, medium, high) and score type.

**Conclusion:**

The EMR-based SEPSIS score shows a similar ability to predict important clinical outcomes compared with other validated scores (qSOFA and NEWS2). Because of the SEPSIS score’s simplicity, it may prove a useful tool for clinical and research applications.

**Disclosures:**

**All Authors**: No reported disclosures

